# Ameliorative Effects of Neutral Electrolyzed Water on Growth Performance, Biochemical Constituents, and Histopathological Changes in Turkey Poults during Aflatoxicosis

**DOI:** 10.3390/toxins9030104

**Published:** 2017-03-14

**Authors:** Denise Gómez-Espinosa, Francisco Javier Cervantes-Aguilar, Juan Carlos Del Río-García, Tania Villarreal-Barajas, Alma Vázquez-Durán, Abraham Méndez-Albores

**Affiliations:** 1 National Autonomous University of Mexico-Superior Studies Faculty at Cuautitlan (UNAM-FESC), Master in Animal Production and Health Sciences, Cuautitlan Izcalli 54714, Mexico; denisegoes1985@gmail.com; 2 National Autonomous University of Mexico-Superior Studies Faculty at Cuautitlan (UNAM-FESC), Veterinary Medicine and Animal Husbandry, Department of Biological and Livestock Sciences, Cuautitlan Izcalli 54714, Mexico; fjca_03@hotmail.com; 3 National Autonomous University of Mexico-Superior Studies Faculty at Cuautitlan (UNAM-FESC), Campus 4, Multidisciplinary Research Unit L14 (Food, Mycotoxins and Mycotoxicosis), Cuautitlan Izcalli 54714, Mexico; delriog@unam.mx (J.C.D.R.-G.); almavazquez@comunidad.unam.mx (A.V.-D.); 4 Esteripharma SA de CV, Atlacomulco 50450, Mexico; tvillarreal@esteripharma.com.mx

**Keywords:** maize, aflatoxins, neutral electrolyzed water, detoxification, turkey

## Abstract

Different in vitro and in silico approaches from our research group have demonstrated that neutral electrolyzed water (NEW) can be used to detoxify aflatoxins. The objective of this investigation was to evaluate the ability of NEW to detoxify B-aflatoxins (AFB_1_ and AFB_2_) in contaminated maize and to confirm detoxification in an in vivo experimental model. Batches of aflatoxin-contaminated maize were detoxified with NEW and mixed in commercial feed. A total of 240 6-day-old female large white Nicholas-700 turkey poults were randomly divided into four treatments of six replicates each (10 turkeys per replicate), which were fed ad libitum for two weeks with the following dietary treatments: (1) control feed containing aflatoxin-free maize (CONTROL); (2) feed containing the aflatoxin-contaminated maize (AF); (3) feed containing the aflatoxin-contaminated maize detoxified with NEW (AF + NEW); and (4) control feed containing aflatoxin-free maize treated with NEW (NEW). Compared to the control groups, turkey poults of the AF group significantly reduced body weight gain and increased feed conversion ratio and mortality rate; whereas turkey poults of the AF + NEW group did not present significant differences on productive parameters. In addition, alterations in serum biochemical constituents, enzyme activities, relative organ weight, gross morphological changes and histopathological studies were significantly mitigated by the aflatoxin-detoxification procedure. From these results, it is concluded that the treatment of aflatoxin-contaminated maize with NEW provided reasonable protection against the effects caused by aflatoxins in young turkey poults.

## 1. Introduction

Among the many types of microorganisms that cause food-borne disease outbreaks, toxigenic fungi and their products (mycotoxins) threaten the health and economy of the poultry industry by contaminating feed materials [1,2,3,4]. Mycotoxins are widespread food and feed compounds capable of causing innumerable damaging effects to humans and animals; these contaminants are a chemical assemblage of fungal-originated toxins that may be found naturally in grains and cereal products. Aflatoxins are a special group of mycotoxins compelling investigation due to their toxic, mutagenic, teratogenic and carcinogenic potential. Some species of fungi of the *Aspergillus* genus, for instance *Aspergillus flavus* and *Aspergillus parasiticus*, produce aflatoxins. These are secondary metabolites easily found in fungal-contaminated peanuts, maize, pistachios and rice, among others, and include four major structures: AFB_1_, AFB_2_, AFG_1_, and AFG_2_ [5,6,7,8]. 

Aflatoxins have been reported to cause severe economic losses in the poultry industry due to their various health effects. Specific responses of aflatoxicosis include reduced growth performance, increased relative pancreas weight, decreased relative liver weight, immunosuppression, hemorrhage, alteration in digestion absorption, nutrient metabolism, serum enzyme activities, biochemical and hematological values and possibly death [9,10]. Specifically, turkeys fed with aflatoxins rapidly incorporate the metabolite in the small intestine, thus mainly damaging the liver. Reduction of protein production, increase in hepatic enzyme activity and coagulopathies indicate fat degeneration and proliferation of biliary ducts [11]. Turkey poults generally exhibit aflatoxicosis as erratic walk, inappetence, reduced activity, anemia, and even death. Therefore, in the poultry industry, production parameters such as body weight gain, feed consumption and mortality rate may be affected by aflatoxin poisoning [12].

Developing strategies to detoxify mycotoxin contamination is crucial for the food and animal feed industries. The best option to prevent mycotoxicosis is to avoid the use of contaminated materials, followed by fungi disinfection [13,14], and finally inactivation of the mycotoxin. A diversified portfolio of strategies to detoxify food and feed has been proposed to reduce the adverse effects of aflatoxin contamination, including physical selection of the contaminated commodity, thermal inactivation, irradiation and supplementation with nano-clay adsorbent, biological degradation by microbial fermentation, and chemical treatment with acids, bases, organic solvents, and gases [1,15]. Many control methods are impractical because they also alter the quality of the food and feed materials; moreover, some of them are expensive and environmental unfriendly. Hence, research into safe and effective alternatives of decontamination has gained much attention recently.

Electrolyzed oxidizing water (EOW) has notable biocidal activity mainly due to three physicochemical properties: pH, oxidation-reduction potential (ORP) and available chlorine concentration (ACC). EOW is produced by electrolysis of pure water added with sodium chloride in an electrolysis apparatus. Water molecules and chloride ions are transformed into chlorine oxidants such as hypochlorous acid (HOCl), hypochlorite ions (ClO^−^) and chlorine (Cl_2_) [16]. During the electrolysis, neutral electrolyzed water (NEW) is generated and it is stable in terms of loss of chlorine oxidants and ORP. Since NEW is non-toxic, non-corrosive and safe due to its physicochemical properties and a reverting capacity to ordinary water when diluted with tap water [17], NEW could be used for the application of safer, healthier and more acceptable methods for aflatoxin detoxification. Mounting evidence suggests that EOW has strong antifungal activity [18,19] and it is also effective in detoxifying AFB_1_ in peanuts and maize [20,21]; however, there are some challenges faced in the development of this technology, such as the quick loss of activity in the presence of organic matter. Nevertheless, the application of NEW to detoxify aflatoxin-contaminated maize and the confirmation of the detoxification process in an in vivo experimental model have not yet been reported. Consequently, the purpose of this study was to evaluate the ability of NEW to detoxify B-aflatoxins in contaminated maize and confirm detoxification in turkey poults.

## 2. Results and Discussion

### 2.1. Physicochemical Properties of NEW

**Figure 1:**
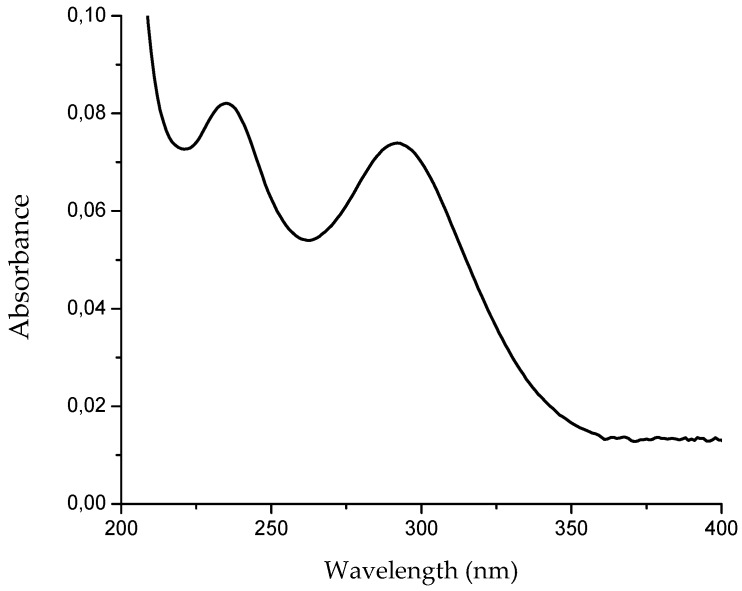
Ultraviolet absorption spectrum of neutral electrolyzed water (NEW) at 25 °C.

Regarding the main physicochemical properties of NEW, after electrolysis and up to the end of the experiment, the pH value (pH 7.03 ± 0.02), ORP (862 ± 3.9 mV) and ACC (54 ± 1 mg/L) were completely stable. It has been established that the most important factor in AFB_1_ transformation is the high level of ACC, which is totally dependent on the electrolysis conditions. Taking into account that NEW contains primarily hypochlorous acid and the hypochlorite ions [16], their concentrations were also determined spectrophotometrically. Ultraviolet spectrum demonstrated two absorption peaks, one at 237 nm and one at 293 nm (Figure 1). Thus, using Lambert-Beer’s law equation, NEW contained 43.01 ± 1.7 mg/L and 10.87 ± 0.71 mg/L of HOCl and OCl^−^, respectively. The Ultra High Range Chlorine photometer uses the *N*,*N*-diethyl-*p*-phenylenediamine ferrous ethylenediammonium sulfate (DPD-FEAS) method, which is a titrimetric procedure for determining free available chlorine; however, care should be taken with this procedure as it cannot differentiate between the HOCl and OCl^−^ concentrations. Consequently, ultraviolet determination could be considered as a practical, inexpensive and environmentally friendly technique for chlorine measurement that allows separate determinations of the two free chlorine species in NEW without the use of chemicals [22].

### 2.2. Aflatoxin Analyses and NEW Detoxifying Capacity

**Figure 2:**
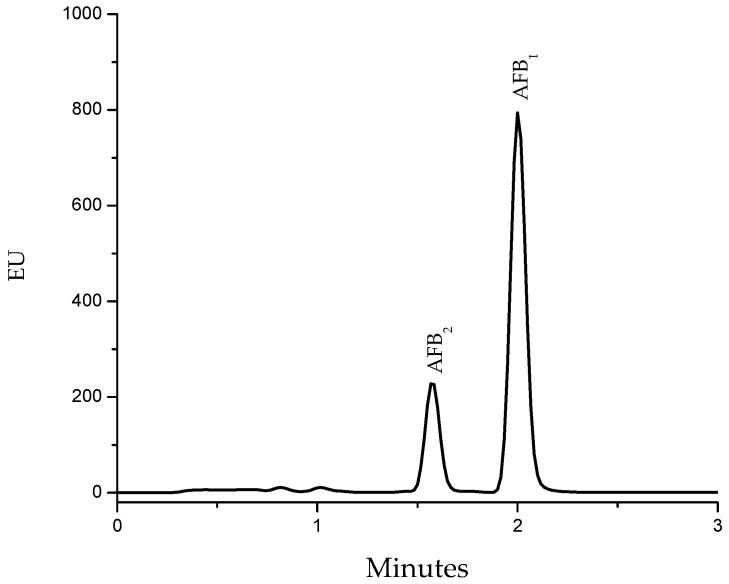
Representative Ultra Performance Liquid Chromatography (UPLC) profile of the aflatoxin-contaminated turkey feed. The retention time values for AFB_2_ and AFB_1_ were 1.60 and 2.00 min, respectively.

The aflatoxin analyses indicated that artificially contaminated feed contained 430 ± 17 ng/g. The Ultra Performance Liquid Chromatography (UPLC) chromatogram confirmed that the toxins were only AFB_2_ and AFB_1_. The retention time (Rt) values for these toxins were 1.60 and 2.00 min, respectively. AFB_1_ was the most abundant toxin, making up 77% (≈331 ng/g) of the total aflatoxin content (Figure 2). In this research, the presence of AFB_2_ in the contaminated feed was considered insignificant, since this molecule is approximately 200-fold less toxic than AFB_1_ [23]. Moreover, no T-2/HT-2 toxins or fumonisins (B_1_, B_2_ and B_3_) were detected in the ration. After treatment with NEW, the aflatoxin content in the detoxified ration did not significantly decrease (determined with both methods via fluorescence detection); in consequence, the aflatoxin fluorescence strength of NEW-detoxified feed was almost similar to the aflatoxin-contaminated feed samples, since it has been reported that this particular detoxification procedure does not reduce the aflatoxin content, measured as loss of fluorescence [20]. Recently, our research group demonstrated that –Cl and –OH groups of the HOCl were added to the C_8_ and C_9_ atoms in the terminal furan ring of the AFB_1_ molecule to yield 8-chloro-9-hydroxy-aflatoxin B_1_. Data of the compound indicate an important reduction in the cytotoxic and genotoxic potential, as demonstrated by using different in vitro and in silico assays [20,24]. The most important factor in AFB_1_ detoxification is the high concentration of HOCl [24]; in this study, NEW contained 43.01 mg/L; however, the post-reaction concentration was <1 mg/L. This reduction represents about 98% decrease in HOCl concentration. In addition, the pH and ORP values were also significantly reduced, reaching values of 5.99 and 232 mV, respectively.

### 2.3. Poult Performance

The effects of providing the aflatoxin-contaminated and NEW-detoxified feed on poult performance and mortality rate are summarized in Table 1. At the end of week 1 (13 days-old), there were no statistical differences in body weight gain among the four treatments. However, by the end of week 2 (20 days-old), body weight gain was significantly reduced in poults of the AF group when compared to the CONTROL and NEW groups, respectively. The poults receiving the aflatoxin-contaminated diet had a 33.6% reduction in body weight gain as compared to the controls. This notable decrement in body weight gain caused by the aflatoxins was significantly ameliorated by the detoxification procedure of the aflatoxins with NEW. Moreover, cumulative data (6 to 20 days) showed that poults of the AF group had −24% deviation in body weight gain in comparison with the CONTROL and NEW groups, respectively. However, a moderate deviation in body weight gain (−11%) was observed in poults of the AF + NEW group, which means that NEW treatment offers a reasonable protection against the harmful effects in body weight gain caused by the aflatoxins. It is also important to mention that performance was not affected by treatment with NEW alone.

It is well known that growth performance of turkey poults can be modified significantly by the presence of aflatoxins in the diet in a dose-dependent manner [4]. McKenzie et al. [2] reported 23% reduction in body weight gain in turkey poults receiving approximately 560 ng/g AFB_1_ in the diet by the end of week 3. Rauber et al. [4] showed growth rate depressions of 16% and 39% in initial-phase turkey poults fed a diet containing 500 ng/g and 1000 ng/g total aflatoxins (AFB_1_ = 75.2%, AFB_2_ = 1.4%, AFG_1_ = 22.6%, AFG_2_ = 0.8%), respectively. The current study demonstrated that a dose of 331 ng/g of AFB_1_ in the diet significantly reduced the body weight gain in turkey poults from 6 to 20 days of age. This finding is in close agreement with the results found by Hamilton et al. [25] and Giambrone et al. [26], who reported that 250 ng/g and 400 ng/g are the minimum contents of AFB_1_ that can significantly affect body weight gain in turkey poults. On the other hand, feed conversion ratio (g feed:g body weight gain) was only affected in poults of the AF group, presenting values up to 2.841 (Table 1). Lala et al. [1] reported a feed conversion rate of 2.930 in starter turkeys fed a diet containing 110 ng/g AFB_1_, which is consistent with our findings. In this investigation, the observed mortality during the 2-week period was as follows: 13 poults in the AF group (22%), 2 poults in the group AF + NEW (3%) and 0 mortality in the CONTROL and NEW groups, respectively. It is important to mention that mortality in the AF group occurred within the first days of the second week of the trial and that the second poult in the AF + NEW group died from chronic staphylococcal peritonitis. The high mortality rate registered in poults of the AF group could be attributed to the formation of the intermediate product 2,3-dihydro-AFB_1_ in the liver, resulting in the rapid onset of necrosis, breakdown of the immune system and eventually death [3]. Rauber et al. [4] reported mortality rates of 18.7% and 37.5% in turkey poults receiving 200 ng/g or 1000 ng/g total aflatoxins. Data shown by these researchers are consistent with our results. Aflatoxin sensibility varies among species, with turkeys being the most sensitive due to a combination of efficient activation and deficient detoxification of this mycotoxin in the liver, the primary target organ [27]. Turkeys that consume aflatoxins in their diet generally develop inappetence, reduced activity, unsteady gait, recumbence and ultimately death [28]. During the experiment, turkey poults of the AF group showed these markedly clinical signs of aflatoxicosis plus poor fathering, apathy and convulsive crises, signs that were completely alleviated by the detoxification treatment with the use of NEW. 

### 2.4. Biochemical Constituents

Table 2 shows the summarized serum biochemical results. In general, aflatoxins caused a significant decrease in the total protein and albumin concentrations; reductions of 35% and 38% in these parameters were observed in poults of the AF group in comparison to the CONTROL group. McKenzie et al. [2] reported decrements of 59% and 53% in the serum concentrations of total protein and albumin in turkey poults fed a diet containing 560 ng/g AFB_1_ during a 3-week period. Quist et al. [12] reported reductions of 19% and 27% in the total protein and albumin concentrations in 4-month old wild turkey poults fed a diet containing 400 ng/g total aflatoxins during 14 days. It has been reported that biochemical changes in the nitrogenous compounds in poults are maximal during the first three weeks after aflatoxin exposure, with hypoalbuminemia and the resultant hypoproteinemia being the most sensitive indicators of aflatoxin intoxication [29]. These effects are compatible with the compromised hepatic function seen in turkey poults of the AF group. Furthermore, an increment of 49% in the creatinine concentration was recorded in poults of the same experimental group (Table 2). Mathuria and Verma [30] reported a dose-dependent increase in creatinine concentration in young inbred Swiss strain male albino mice administered with aflatoxins for a 45-day period. Authors reported that low doses of aflatoxins (750 µg/kg body weight) caused a 60% increment in the creatinine concentration as compared to the vehicle control. The heightened appearance of creatinine in the serum of poults in the AF group indicates the increased transformation of phosphocreatinine to creatinine in the muscle, which might be due to a lesser utilization of phosphocreatinine during muscular contraction. Since the kidney rapidly excretes creatinine, the significant increase in creatinine concentration in the serum could be due to increased release from muscles and/or decreased excretion from the kidney, suggesting that aflatoxins cause adverse changes in both skeletal muscle and kidney at very early stages [30]. Furthermore, there were no significant differences in total bilirubin and uric acid concentrations among all four treatments (Table 2). In general, it can be concluded that the detoxification procedure with the use of NEW ameliorates aflatoxin-induced effects in the serum parameters as compared to the aflatoxin alone treated group.

### 2.5. Serum Enzyme Activities

Conspicuous symptoms of aflatoxin toxicity were detected in poults of the AF group by the serum aspartate aminotransferase (AST) activity level, which increased by 1.34-fold in comparison to the CONTROL group (Table 3). On the other hand, there were no significant differences in alanine aminotransferase (ALT), gamma glutamiltranspeptidase (GGT) and alkaline phosphathase (AP) activities among all four treatments, which is in close agreement with data reported by Rauber et al. [4] in turkey poults fed seven different doses of aflatoxins in their diet. It is well known that AST is present in the cytosol, and whenever liver hepatocytes are damaged, the enzyme is released into the blood stream; consequently, a significant increase in its activity indicates damage. The results obtained in the present study showed a significant increase in the AST activity in poults of the AF group; similar results have been reported by different researchers [2,12,31]. However, this effect was completely mitigated by the treatment of the aflatoxin molecules with NEW. 

### 2.6. Relative Organ Weight

As shown in Table 4, no significant differences were noted among all treatments in the relative spleen and bursa of Fabricius relative weights. However, when compared to the CONTROL group, the relative weight of the liver decreased significantly (20%), whereas the relative weight of the kidney increased (37%), in poults of the AF group. The reduced relative liver weight and the increments in the relative kidney weight in poults of the AF group are consistent with previous studies [2,4,12]. In general, the aflatoxin detoxification procedure with the use of NEW protected the poults from significant relative organ weight changes. 

### 2.7. Gross Morphological Changes and Histopathological Studies

**Figure 3:**
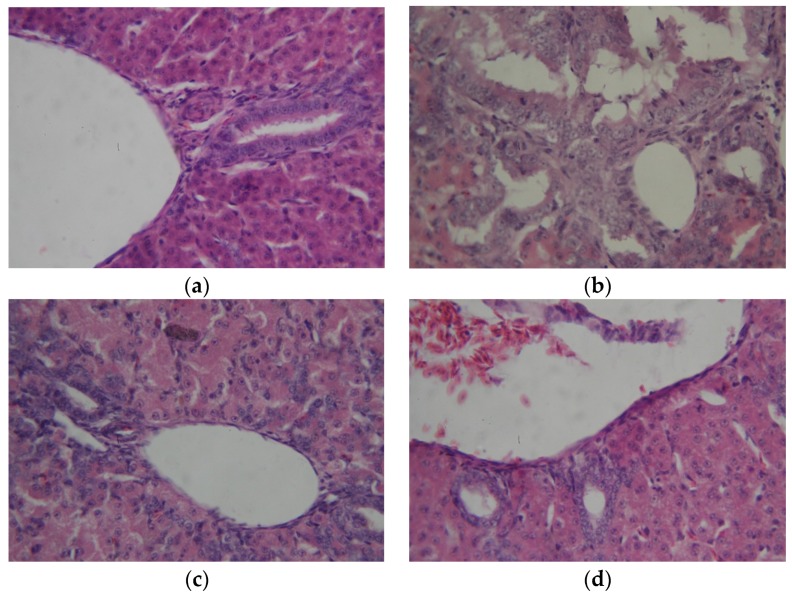
Comparative histological changes in the liver (20×, H & E stain). The normal histological structure is similar in the CONTROL (profile **a**) and NEW (profile **d**) groups. Massive bile duct epithelial necrosis as well as severe hyperplasia of the epithelium and bile duct proliferation is observed in the AF group (profile **b**), in contrast with a minimal degree of bile duct epithelial necrosis and minimal bile duct proliferation seen in the AF + NEW group (profile **c**).

**Figure 4:**
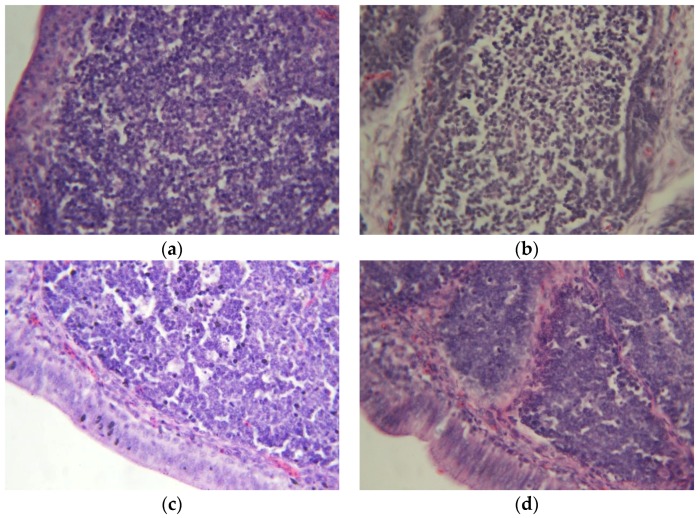
Comparative histological changes in the bursa of Fabricius (20×, H & E stain). The normal structure is similar in the CONTROL (profile **a**) and NEW (profile **d**) groups. Severe lymphoid depletion and hyperplasia of the epithelium is clear in the AF group (profile **b**), in comparison with minimal degree of lymphoid depletion and hyperplasia of the epithelium in the AF + NEW group (profile **c**).

**Figure 5:**
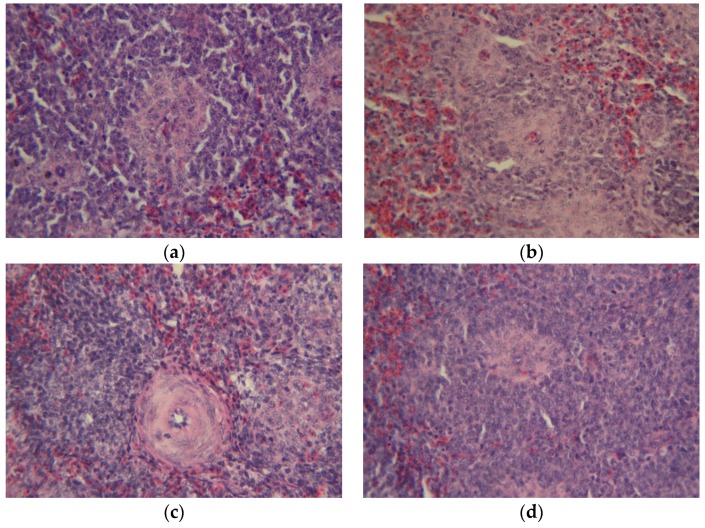
Comparative histological changes in the spleen (20×, H & E stain). The normal histological structure in the CONTROL (profile **a**) is similar to that of group NEW (profile **d**). Severe and minimal lymphoid depletion was observed in the AF and AF + NEW groups (profiles **b**,**c**), respectively.

No gross morphological alterations were observed in the liver, kidney, spleen or bursa of Fabricius of the poults in the CONTROL, AF + NEW and NEW groups throughout the course of the experiment. However, livers of the AF group were pale, friable and appeared smaller in size compared to those of the CONTROL group. These lesions were also accompanied by hemorrhagic streaks and accentuated lobular patterns. On the other hand, the AF group kidneys showed no gross lesions but were swollen and had light colored areas on the surface. Furthermore, histological studies provided additional evidence of the beneficial effect of NEW treatment on suppressing the toxicity induced by the aflatoxins. In general, aflatoxin exposure caused extensive liver damage in all poults of the AF group; lesions observed were composed of variable areas of hepatic degeneration, massive bile duct epithelial necrosis as well as severe hyperplasia of the epithelium and bile duct proliferation (Figure 3, profile b). In contrast, only a minimal degree of bile duct epithelial necrosis and minimal bile duct proliferation was seen in the livers of AF + NEW group (Figure 3, profile c). Histopathological examination of the bursa of Fabricius indicated lymphoid depletion, abundant apoptotic figures in the germinal centers and hyperplasia of the epithelium—all of them in severe grade—in poults of the AF group (Figure 4, profile b). On the contrary, only a minimal degree of lymphoid depletion and hyperplasia of the epithelium was seen in the bursa of Fabricius of poults of the AF + NEW group (Figure 4, profile c). Lesions on the spleen were more discrete; the changes seen in poults of the AF and AF + NEW groups, but not seen in the CONTROL and NEW groups, were severe and minimal lymphoid depletion, respectively (Figure 5, profiles b,c). Finally, no significant pathological changes on the kidney were found in poults of the four experimental groups (not shown). It has been reported that the principal target organ for aflatoxicosis is the liver; consequently, all microscopic lesions caused by the aflatoxins agree with our previous studies [32]. On the contrary, the severity of lesions in the liver was significantly reduced in the AF + NEW treatment group, suggesting that the detoxification procedure with the use of NEW is effective in reducing the toxicity of aflatoxins in the liver of turkey poults. Aflatoxins are also potent immunosuppressive agents causing immunosuppression through impairment of humoral and cellular immune responses [26]; thus, resistance to infectious diseases is generally dependent on antibody production which is dependent in turn on the bursa of Fabricius. In this research, aflatoxins caused severe depletion of lymphoid cells in the bursa of Fabricius and spleen, indicating that lymphoid organs are very sensitive to aflatoxins. However, similar but less severe changes in the bursa of Fabricius and spleen were seen in turkey poults of the AF + NEW group when compared with the AF group. These findings provide further evidence of the effectiveness of the detoxification treatment in reduction of aflatoxin toxicity. 

To the best of our knowledge, the present study is the first one to demonstrate that treatment of aflatoxin-contaminated maize with NEW significantly diminishes AFB_1_-toxicity in young turkey poults. This effect can be related to the reaction of hypochlorous acid with the double bond in the terminal furan ring of the AFB_1_ molecule to yield 8-chloro-9-hydroxy-aflatoxin B_1_. Nevertheless, further studies on the complete growing cycle of other animal species need to be conducted. Finally, to accurately determine the risk of using chlorine compounds in the animal feed industry, the available chlorine concentration used and the reactivities of the chlorine species with feed components such as carbohydrates, lipids, and proteins should be extensively evaluated.

## 3. Materials and Methods 

### 3.1. Safety Precautions

Procedures used for handling and decontaminating aflatoxin-contaminated materials were adopted from recommendations published by the International Agency for Research on Cancer [33].

### 3.2. Animal Ethics

This study was conducted according to the Internal Committee for Care and Use of Experimental Animals (CICUAE), approved by the National Autonomous University of Mexico. Ethical approval code: CICUAE-29102009. Date of approval: 29 October 2009. Project identification code: C16_02. Date of approval: 8 April 2016. Ethics committee: Internal Committee for Care and Use of Experimental Animals (CICUAE, from its abbreviation in Spanish).

### 3.3. Chemicals and Reagents

B-aflatoxins, tween-80, sodium chloride, formaldehyde, xylenes and hematoxylin-eosin staining solution were purchased from Sigma-Aldrich Co. (St. Louis, MO, USA). Acetonitrile (HPLC grade), ethanol, methanol, and methanol (HPLC grade) were obtained from J.T Baker, Mallinckrodt Baker (Ecatepec, Mexico). Paraffin for tissue embedding was procured from Leica Biosystems Richmond Inc. (Richmond, IL, USA). Malt extract-sodium chloride-agar (MSA) medium was obtained from DIBICO SA de CV (Mexico, DF, Mexico). Saline solution was purchased from Becton Dickinson Co. (Mexico, DF, Mexico). All other chemicals used were analytical reagent grade.

### 3.4. Preparation of Neutral Electrolyzed Water (NEW)

NEW was electrochemically generated using two patented generators from Esteripharma SA de CV (Atlacomulco, Mexico). This method of NEW production has been previously described in detail by Jardon-Xicotencatl et al. [20]. NEW was used immediately after elaboration. Three physicochemical properties were verified: the pH and ORP values were measured with a combination pH/ORP/temperature meter PC-45 (Conductronic, Mexico, DF, Mexico), and the ACC was determined using an Ultra High Range Chlorine photometer model HI96771C (Hanna Instruments, Melrose, MA, USA). The concentrations of HOCl (the main available chlorine form in NEW) and OCl^−^ were further determined using a diode-array system spectrophotometer Cary-8454 (Agilent Technologies, Santa Clara, CA, USA). Ultraviolet spectra was collected in the range of 200–400 nm at room temperature in 1-cm quartz cell and the absorbance at 237 nm and 293 nm was used to determine the concentration using Lambert-Beer’s law equation with 100 1/M·cm and 350 1/M·cm as the molar absorption coefficients for HOCl and OCl^−^, respectively [34].

### 3.5. Maize Grain

White maize grains of the commercial hybrid AS-900 provided by Aspros Comercial SA de CV (Cortazar, Mexico) were used. The aflatoxin content of the grain was below the immunoaffinity column minimum detection limit (0.5 ng/g). Contents of T-2/HT-2 toxins and total fumonisins (FB_1_, FB_2_ and FB_3_) were also determined using monoclonal antibody columns [35]. Moisture content was determined by drying replicate portions of 5 to 10 g each of whole grain at 103 °C for 72 h, with percentages calculated on a wet-weight basis. 

### 3.6. Fungal Isolate

The *Aspergillus flavus* Link strain UNIGRAS-1231 (Culture Collection of the Grain and Seed Research Unit of the National Autonomous University of Mexico) was plated into Petri dishes containing MSA medium (%: malt extract, 2; sodium chloride, 6; and agar, 2) for 7 days at 25 °C. This strain has high ability to produce AFB_1_ and AFB_2_ [36].

### 3.7. Aflatoxins Production

Aflatoxins were produced according to the technique proposed by Méndez-Albores et al. [36]. The inoculated maize was incubated during 37 days to obtain an approximate aflatoxin content of 13,500 ng/g. The aflatoxin-contaminated maize was stored at 4 °C prior to treatment.

### 3.8. Aflatoxin Analyses

#### 3.8.1. Using Immunoaffinity Columns (IACs)

The aflatoxin content was determined according to the 991.31 AOAC method [37] using antibody-based IACs for AFB_1_ and AFB_2_. Samples (50 g) were extracted by blending with 100 mL methanol-water (80:20, *v*/*v*) and 5 g of NaCl using a laboratory blender model 51BL30 (Waring, CT, USA). The mixture was filtered through a Whatman 1 filter paper and a portion of 5 mL was diluted with 20 mL of distilled water. The diluted preparation was filtered through a micro-fiber filter, and 10 mL were passed through the IACs (Afla B, VICAM Science Technology, Watertown, MA, USA). Subsequently, the column was washed twice with 10 mL of distilled water and dried with sterile air flow. The toxins were then eluted with 1 mL of HPLC grade methanol and quantified in a fluorometer VICAM Series-4EX (VICAM Source Scientific, Irvine, CA, USA) after reaction with 1 mL of 0.002% aqueous bromine. The detection limit for aflatoxins via fluorescence measurement is approximately 0.5 ng/g. 

#### 3.8.2. Using Ultra Performance Liquid Chromatography (UPLC)

The aflatoxin identification was carried out according to the technique proposed by Jardon-Xicontencatl et al. [20], using a Waters ACQUITY UPLC H-Class System equipped with a quaternary solvent manager and an ACQUITY UPLC BEH C18 phase reverse column (2.1 × 100 mm, 1.7 µm). Standards, as well as samples collected from the IACs (1 µL), were injected and eluted with a single ternary mixture of 64:18:18 water/methanol/acetonitrile (all HPLC grade) at a flow rate of 400 µL/min. Aflatoxins were fluorometrically detected and identified using a UPLC-optimized fluorescence detector (Waters, Milford, MA, USA). The excitation and emission wavelengths were 365 and 429 nm, respectively. Aflatoxins were identified by their Rt and compared with those of a pure aflatoxin standard solution. The estimated detection limits are 0.58 and 2.01 ng/kg for AFB_2_ and AFB_1_, respectively. 

### 3.9. Detoxification of the Aflatoxin-Contaminated Maize with NEW and Diet Formulation

Batches of the aflatoxin-contaminated maize (1 kg) were milled (Molinos Pulvex, Mexico, DF, Mexico) using a hammer head and a 0.5 mm mesh screen, and subjected to detoxification treatment using standardized conditions previously outlined by Jardon-Xicotencatl et al. [20]. Subsequently, the aflatoxin-detoxified material was placed into flat stainless steel pans for 2 h in a sterile air flow chamber. The ground maize was mixed in a commercial turkey feed procured from Nutricion Tecnica Animal SA de CV (Queretaro, Mexico) containing 26% protein, 12.64 MJ/kg (metabolizable energy) and met or exceeded levels of other nutrients recommended by the National Research Council [38]. The commercial feed was free of any detectable mycotoxins (aflatoxins, T-2/HT-2 toxins and fumonisins) and contained no antibiotic, nor anticoccidial drugs or even growth promoters. Feed batches (25 kg) were artificially contaminated with B-aflatoxins (430 ng/g) using 27 g of the milled maize per kilogram of feed. In order to assure the proper distribution of the aflatoxins, the contaminated feed was mixed for 20 min in a Ribbon Blender Mixer model MH-7050 (Molinos Pulvex, Mexico, DF, Mexico).

### 3.10. Birds and Housing

For the experiment, 240 6-day-old female large white Nicholas-700 turkey poults (obtained from a commercial hatchery) were individually weighed at placement and randomly distributed in four pens at the Poultry Research Station of the National Autonomous University of Mexico. Six replicates of ten poults (*n* = 60 per treatment) were grouped based on the following four dietary treatments: (1) control feed containing aflatoxin-free maize (CONTROL); (2) feed containing the aflatoxin-contaminated maize (AF); (3) feed containing the aflatoxin-contaminated maize detoxified with NEW (AF + NEW); and (4) control feed containing aflatoxin-free maize treated with NEW (NEW). The temperature, lighting and ventilation programs were followed according to standard recommendations of the supplier. Feed and water were provided ad libitum during the whole period of the experiment (2 weeks).

### 3.11. Collection of Samples and Measurements

Poults were individually weighed (on a weekly basis) and feed consumption for each replicate was measured weekly until the end of the experiment (2 weeks). Body weight gain, feed intake, feed conversion ratio (FCR) and mortality rate (MR) were calculated. Feed intake and FCR were adjusted for mortalities when necessary. At 20 days of age, blood was drawn by cardiac puncture under anesthesia (the turkey was exposed for 1 minute to 40% carbon dioxide, 30% oxygen, and 30% nitrogen) from 18 randomly selected birds from each treatment (3 birds per replicate), and serum was prepared. The following analyses were performed spectrophotometrically using commercially available kits (BioSystems, Barcelona, Spain): total protein (545 nm), albumin (630 nm), total bilirubin (540 nm), creatinine (500 nm), and uric acid (520 nm). The serum aspartate aminotransferase (AST), alanine aminotransferase (ALT), gamma glutamiltranspeptidase (GGT), and alkaline phosphathase (AP) activities were also determined spectrophotometrically at 340 nm, 340 nm, 410 nm and 405 nm, respectively. The bled poults were then exposed to 80% carbon dioxide, 5% oxygen, and 15% nitrogen for euthanasia [39]. All efforts were made to minimize unnecessary pain and suffering. Liver plus gall bladder, kidney, spleen, and bursa of Fabricius were excised and washed in cold saline and their relative percentages estimated. For histopathological examination, all specimens were fixed in 10% neutral-buffered formalin for 48 h, routinely embedded in paraffin, cut into 4 µm thick sections and processed for hematoxylin and eosin (H & E) staining.

### 3.12. Statistical Analysis

Data were subjected to analysis of variance (ANOVA) using the General Linear Model (GLM) procedure in Statistical Analysis System software version 8.0 [40], and means were separated by the Dunnett procedure and judged to be significantly different if *p* < 0.05.

## Figures and Tables

**Table 1:** Effects of aflatoxin-contaminated and NEW-detoxified feed on body weight gain, feed conversion ratio and mortality rate in turkey poults.

Treatment	Body Weight Gain (g)			Deviation from CONTROL (%)	FCR (g Feed: g Gain)	MR (%)
	6 to 13 days old	13 to 20 days old	6 to 20 days old			
CONTROL	51.3 ± 4.2 ^a^	131.9 ± 4.5 ^a^	183.2	0	2.021 ^a^	0
AF	52.2 ± 1.9 ^a^	87.6 ± 5.8 ^b^	139.8	−24	2.841 ^b^	22
AF + NEW	50.5 ± 3.3 ^a^	111.8 ± 7.5 ^a^	162.3	−11	2.184 ^a^	3
NEW	52.2 ± 3.0 ^a^	131.8 ± 11.9 ^a^	184.0	0	2.149 ^a^	0

Mean of six replicates of ten poults each per treatment (minus mortality) ± standard error. ^a,b^ Means, within the same column, not sharing a common superscript differ significantly (Dunnett test *p* < 0.05). FCR = feed conversion ratio, MR = mortality rate.

**Table 2:** Effects of aflatoxin-contaminated and NEW-detoxified feed on some biochemical constituents in turkey poults at the end of the trial.

Treatment	Total Protein	Albumin	Total Bilirubin	Creatinine	Uric Acid
	(g/dL)		(mg/dL)		
CONTROL	2.42 ± 0.64 ^a^	0.69 ± 0.16 ^a^	0.28 ± 0.32 ^a^	0.35 ± 0.04 ^a^	6.98 ± 1.88 ^a^
AF	1.57 ± 0.07 ^b^	0.43 ± 0.02 ^b^	0.22 ± 0.15 ^a^	0.52 ± 0.10 ^b^	4.09 ± 0.92 ^a^
AF + NEW	1.97 ± 0.33 ^a^	0.61 ± 0.23 ^a^	0.25 ± 0.04 ^a^	0.35 ± 0.12 ^a^	4.24 ± 0.17 ^a^
NEW	2.38 ± 0.51 ^a^	0.79 ± 0.09 ^a^	0.25 ± 0.19 ^a^	0.39 ± 0.08 ^a^	6.26 ± 1.70 ^a^

Mean of six replicates of three poults each per treatment (*n* = 18) ± standard error. ^a,b^ Means, within the same column, not sharing a common superscript differ significantly (Dunnett test *p* < 0.05).

**Table 3:** Effects of aflatoxin-contaminated and NEW-detoxified feed on serum enzyme activities in turkey poults at the end of the trial.

Treatment	AST	ALT	GGT	AP
	(IU/L)			
CONTROL	478.00 ± 8.49 ^a^	49.55 ± 3.66 ^a^	7.60 ± 0.75 ^a^	2360.91 ± 26.35 ^a^
AF	644.32 ± 5.60 ^b^	54.82 ± 9.83 ^a^	7.60 ± 0.61 ^a^	2136.66 ± 59.52 ^a^
AF + NEW	369.01 ± 2.50 ^a^	67.32 ± 5.55 ^a^	6.66 ± 0.78 ^a^	2189.73 ± 33.80 ^a^
NEW	536.44 ± 7.05 ^a^	50.60 ± 7.17 ^a^	9.67 ± 0.42 ^a^	2481.40 ± 85.24 ^a^

Mean of six replicates of three poults each per treatment (*n* = 18) ± standard error. ^a,b^ Means, within the same column, not sharing a common superscript differ significantly (Dunnett test *p* < 0.05). AST = aspartate aminotransferase, ALT = alanine aminotransferase, GGT = gamma glutamiltranspeptidase, AP = alkaline phosphathase.

**Table 4:** Effects of aflatoxin-contaminated and NEW-detoxified feed on relative organ weight in turkey poults at the end of the trial.

Treatment	Liver	Kidney	Spleen	Bursa of Fabricius
CONTROL	3.18 ± 0.27 ^a^	1.02 ± 0.12 ^a^	0.09 ± 0.01 ^a^	0.18 ± 0.03 ^a^
AF	2.55 ± 0.10 ^b^	1.40 ± 0.07 ^b^	0.08 ± 0.01 ^a^	0.14 ± 0.02 ^a^
AF + NEW	2.91 ± 0.08 ^a^	1.07 ± 0.04 ^a^	0.08 ± 0.01 ^a^	0.14 ± 0.02 ^a^
NEW	3.16 ± 0.04 ^a^	0.97 ± 0.05 ^a^	0.08 ± 0.01 ^a^	0.14 ± 0.01 ^a^

Mean of six replicates of three poults each per treatment (*n* = 18) ± standard error. ^a,b^ Means, within the same column, not sharing a common superscript differ significantly (Dunnett test *p* < 0.05).
